# IDNet: A Diffusion Model-Enhanced Framework for Accurate Cranio-Maxillofacial Bone Defect Repair

**DOI:** 10.3390/bioengineering12040407

**Published:** 2025-04-11

**Authors:** Xueqin Ji, Wensheng Wang, Xiaobiao Zhang, Xinrong Chen

**Affiliations:** 1 The Third School of Clinical Medicine, Ningxia Medical University, Yinchuan 750000, China; jinian54@163.com; 2 Department of Ultrasound, Peking University First Hospital Ningxia Women and Children’s Hospital, Yinchuan 70000, China; 3 Fudan University Academy for Engineering and Technology, Shanghai 200000, China; 22210860059@m.fudan.edu.cn; 4 Shanghai Key Laboratory of Medical Imaging Computing and Computer Assisted Intervention, Shanghai 200000, China; 5 Department of Neurosurgery, Zhongshan Hospital, Fudan University, 180 Fenglin Road, Shanghai 200000, China

**Keywords:** cranio-maxillofacial surgery, bone defect repair, diffusion model, deep learning, medical image segmentation, 3D reconstruction

## Abstract

Cranio-maxillofacial bone defect repair poses significant challenges in oral and maxillofacial surgery due to the complex anatomy of the region and its substantial impact on patients’ physiological function, aesthetic appearance, and quality of life. Inaccurate reconstruction can result in serious complications, including functional impairment and psychological trauma. Traditional methods have notable limitations for complex defects, underscoring the need for advanced computational approaches to achieve high-precision personalized reconstruction. This study presents the Internal Diffusion Network (IDNet), a novel framework that integrates a diffusion model into a standard U-shaped network to extract valuable information from input data and produce high-resolution representations for 3D medical segmentation. A Step-Uncertainty Fusion module was designed to enhance prediction robustness by combining diffusion model outputs at each inference step. The model was evaluated on a dataset consisting of 125 normal human skull 3D reconstructions and 2625 simulated cranio-maxillofacial bone defects. Quantitative evaluation revealed that IDNet outperformed mainstream methods, including UNETR and 3D U-Net, across key metrics: Dice Similarity Coefficient (DSC), True Positive Rate (RECALL), and 95th percentile Hausdorff Distance (HD95). The approach achieved an average DSC of 0.8140, RECALL of 0.8554, and HD95 of 4.35 mm across seven defect types, substantially surpassing comparison methods. This study demonstrates the significant performance advantages of diffusion model-based approaches in cranio-maxillofacial bone defect repair, with potential implications for increasing repair surgery success rates and patient satisfaction in clinical applications.

## 1. Introduction

Oral and cranio-maxillofacial surgery (OCMS) is a specialized field that addresses lesions and deformities in the oral cavity, jaws, face, head, and neck regions [1]. These areas are characterized by complex anatomy, critical physiological functions, and high aesthetic demands. Congenital anomalies, trauma, tumor removal, and infections can lead to significant defects and deformities in the craniomaxillofacial (CMF) region, particularly bone defects, which not only impair normal function and facial appearance but also have profound psychological and social impacts on patients. To address these challenges, precise preoperative planning and minimally invasive surgical techniques are essential. The integration of digital technologies, such as 3D reconstruction, Computer-Aided Design/Computer-Aided Manufacturing (CAD/CAM), and 3D printing, has revolutionized the field, enabling more accurate and personalized treatments. However, traditional methods for defect repair, such as mirroring the healthy side of the jaw to the defect area, are limited by anatomical asymmetries, which are expected to be solved by new advanced solutions, such as artificial intelligence (AI).

The repair of cranio-maxillofacial bone defects is governed by complex biological mechanisms, primarily through the processes of intramembranous and endochondral ossification. Intramembranous ossification, which predominates in the craniofacial skeleton, involves the direct differentiation of mesenchymal stem cells into osteoblasts that deposit osteoid matrix, which subsequently undergoes mineralization to form new bone tissue. This process is particularly prevalent in the flat bones of the cranium and facial structures. In contrast, endochondral ossification proceeds through an intermediate cartilaginous template prior to bone formation and is more commonly observed in load-bearing regions.

The natural repair processes are orchestrated by a complex interplay of cellular activities, growth factors, and biomechanical stimuli. Key signaling molecules, including bone morphogenetic proteins (BMPs), transforming growth factor-*β* (TGF-*β*), and vascular endothelial growth factor (VEGF), play a crucial role in regulating cell migration, proliferation, and differentiation during the repair process. Furthermore, the spatial organization of the extracellular matrix provides essential guidance cues that facilitate tissue regeneration and integration. The computational diffusion model-enhanced framework exhibits conceptual similarities to biological processes. Like natural bone repair, which progresses from an initially disorganized state to a highly structured tissue through coordinated cellular activities and chemical gradients, the diffusion model utilizes a progressive denoising approach to reconstruct anatomical structures via iterative refinement. The step-by-step nature of the diffusion process parallels the temporal progression of biological repair, whereas the Step-Uncertainty Fusion module conceptually resembles the integration of multiple signaling pathways that guide tissue regeneration across different spatial scales.

Insights into these biological principles have informed several design choices in the computational framework, particularly with regards to handling complex geometries and regional variations characteristic of the cranio-maxillofacial skeleton. By incorporating this biomimetic perspective, the reconstruction approach aims to enhance anatomical accuracy and clinical relevance.

With the development of deep learning, digital technology with AI has been rapidly applied in CMF conditions. For instance, convolutional neural networks (CNNs) were used to regress anatomical landmarks on cone-beam computed tomography (CBCT) images and detect jaw cysts or tumors in panoramic X-rays [2]. AI can also assist in diagnosing bony jaw deformities by analyzing cephalometric data [3]. Despite these advances, the application of AI in automatic repair of CMF bone defects is still limited, mainly due to the anatomical complexity and variability of CMF regions [4,5,6], as well as racial and regional differences in bone morphology, which further complicate the development of effective AI models [7,8].

AI-driven algorithms can leverage high-resolution data to create accurate reconstructions [9], adapting to real-world scenarios through training on databases of healthy skulls with simulated defects [10,11,12]. This approach overcomes the limitations of manual contouring, which is subjective, time-consuming, and prone to errors due to anatomical asymmetries [13].

**Figure 1:**
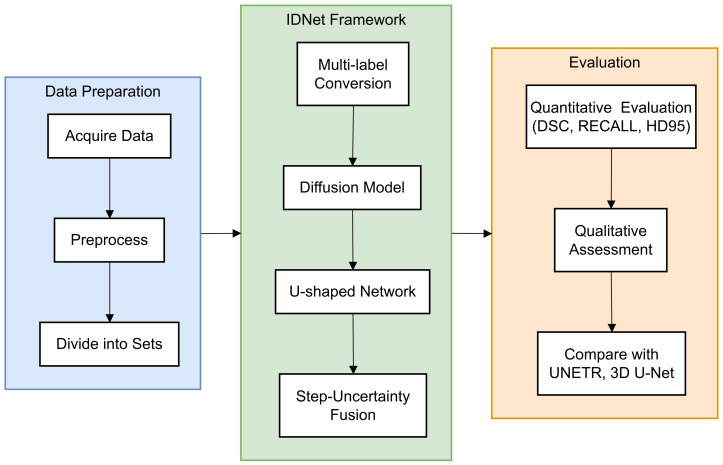
Workflow of the proposed IDNet framework for cranio-maxillofacial bone defect repair. The process begins with dataset acquisition and preprocessing (**left**), proceeds through the IDNet architecture with its key components—multi-label conversion, information extraction unit, and step-uncertainty fusion strategy (**middle**), and culminates in quantitative and qualitative evaluation (**right**). Each component is represented with its specific input–output relationship to provide a clear understanding of the information flow throughout the system.

In this paper, a novel end-to-end framework is designed for 3D volumetric segmentation of medical images [14]. To extract valuable information from the input data and produce high resolution, a diffusion model is integrated into a U-shaped network structure. To enhance the robustness of the diffusion model predictions [15], a Step-Uncertainty Fusion (SUFusion) module is proposed, which combines the diffusion model outputs at each step of the inference process [16]. A comprehensive flowchart (Figure 1) has been added to illustrate the step-by-step workflow of the proposed framework.

## 2. Related Work

The field of computer vision-assisted cranial bone repair has evolved significantly over the years. Initially, approaches relied heavily on hand-designed features and geometric modeling techniques, which entailed complex pre-processing and post-processing steps and had limited effectiveness for complex-shaped cranial repairs [17]. A notable study by Chen et al. [17] proposed a geometrically informed cranial repair method, highlighting the importance of human–computer interaction in the design process. By automating the design of cranial bone repair through mirroring symmetry points and 3D model reconstruction, they successfully reduced surgical time and cost. Sanan and Haines [18] provided a comprehensive review of the history of cranial repair surgery, tracing its development from simple cranioplasty to modern computer-aided design and manufacturing techniques. While acknowledging the effectiveness of traditional methods in certain cases, they noted that these approaches often lack precision and reproducibility. In a review of image restoration techniques, Elharrouss et al. [19] emphasized the significance of automated methods in reducing the physician’s workload and increasing the accuracy and efficiency of the repair. This highlights the need for advanced computational methods in cranial bone repair.

The advent of deep learning techniques has led researchers to leverage convolutional neural networks (CNNs) to automatically capture the intricate structure of the skull. Notably, Preda et al. [20] employed a deep convolutional network to segment cranial bone images automatically, resulting in a significant improvement in repair accuracy. Their study underscores the potential of deep learning in medical image segmentation, particularly in cranial bone repair. In a related study, Jin et al. [21] enhanced the resolution of cranial implant prediction through patch-wise training. This approach enables high-precision reconstruction of cranial details by focusing on localized regions. Kodym et al. [22] explored the application of cascaded CNNs for cranial defect reconstruction, highlighting the importance of alignment and feature fusion in cranial bone repair. The cascade network structure improves the accuracy and robustness of the repair.

The incorporation of the attention mechanism has been shown to further enhance the performance of deep learning models in cranial bone repair tasks. Specifically, Resmi et al. [23] demonstrated a significant improvement in repair accuracy by introducing an attention module that allows the network to selectively focus on critical regions of the skull. This study highlights the crucial role of the attention mechanism in enhancing the model’s ability to recognize key features. In a related study, Wang et al. [24] explored a deep learning approach that integrates anatomical laws for designing cranial implants. Their findings underscore the importance of incorporating anatomical knowledge to achieve improved repair outcomes [25,26].

Recently, diffusion modeling has emerged as a promising generative model, demonstrating its capabilities in various fields. Notably, Jaramillo and Sipiran [27] examined the application of conditional diffusion modeling for the 3D reconstruction of cultural heritage objects. Their findings provide evidence of the potential of diffusion modeling in cultural heritage reconstruction, which may also be relevant to cranial bone repair. In a related study, Wolleb et al. [28] explored the use of diffusion models in implicit image segmentation. This study offers a new perspective on the application of diffusion modeling in medical image processing, with particular implications for the field of cranial bone repair.

## 3. Materials and Methods

### 3.1. CMF Dataset

#### 3.1.1. Data Acquisition

The dataset used in this study consisted of head CT scans from normal adults who attended the Ninth People’s Hospital of Shanghai Jiaotong University School of Medicine between 2008 and 2022. The inclusion criteria were as follows: age between 18 and 60 years, no gender restrictions, whole-head CT scanning range, complete craniofacial bone morphology with no defects or obvious deformities, and no visible surgical traces. Conversely, the exclusion criteria comprised cases with structural defects or incomplete CT 3D reconstruction models, patients exhibiting obvious opening or mandibular movement on the CT scan, abnormal occlusion of the maxillary and mandibular teeth, and those with evident intraosseous diseases such as osteomyelitis or fibrous anomalous proliferative disorder of the jaws, which hindered the construction of the bone defect. Furthermore, cases with rough, noisy, or unsmooth surfaces on the 3D reconstruction model were also excluded. A total of 125 cases of craniofacial CT data were collected for this study.

#### 3.1.2. Structure of Dataset

The dataset comprises 125 3D reconstructions of normal human skulls, along with 2625 simulated craniofacial bone defects. These data were classified into seven distinct categories of defects, namely anterior cranial and frontal, zygomatic arch, zygomatic bone, nasal–orbital sieve (NOE), maxillary sinus wall, mandibular body, mandibular branch, and condyle. Furthermore, each defect was sub-classified according to its location as left, right, or bilateral (i.e., crossing the facial midline).

#### 3.1.3. Data Processing

To simulate the skull of a patient with real defects, 21 distinct defects were created on each 3D reconstructed skull using 3D Slicer software. Morphological repair of the defects was achieved by subtracting combinations of randomly deformed spheres from the edges. The CT data were annotated by a team of doctors and students from Shanghai Jiao Tong University School of Medicine and Fudan University. The images underwent preprocessing, which involved registration alignment, threshold filtering, and preliminary cropping, using Advanced Normalization Tools (ANTs).

### 3.2. IDNet

This paper introduces a novel end-to-end framework, termed Internal Diffusion Network (IDNet), designed for 3D volumetric segmentation of medical images. By integrating a diffusion model into a standard U-shaped network structure, the framework aims to extract valuable information from the input data and produce high-resolution, pixel-level representations suitable for 3D medical segmentation. To enhance the robustness of the diffusion model predictions, a Step-Uncertainty Fusion (SUFusion) module is proposed, which combines the diffusion model outputs at each step of the inference process. An evaluation conducted on the CMF dataset for cranial bone repair demonstrates that the embedded diffusion network significantly outperforms existing techniques in terms of performance. Furthermore, the experimental results showcase the generalizability and effectiveness of the proposed model, highlighting its potential to facilitate accurate diagnosis and treatment of medical conditions by enabling more precise segmentation of anatomical structures.

#### 3.2.1. Internal Diffusion Networks Architecture Specifications

IDNet employs a hierarchical structure with 4 levels of processing. At each level, the network consists of a sequence of 3 residual blocks, where each residual block contains two 3D convolutional layers with 3 × 3 × 3 kernels followed by instance normalization and SiLU activation functions. The number of feature channels starts at 64 in the first level and doubles with each subsequent level (128, 256, and 512 for levels 2, 3, and 4, respectively).

The time embedding dimension for the diffusion process is set to 256, and the time embeddings are processed through a 2-layer MLP with SiLU activations before being injected into each residual block via adaptive instance normalization. For the condition embeddings that incorporate defect region information, a dimension of 128 is utilized, which undergoes processing through a similar MLP structure prior to integration. Skip connections are implemented between corresponding levels in the encoder and decoder paths, with concatenation operations followed by 1 × 1 × 1 convolutional layers to manage feature dimensionality. The final output layer consists of a 3 × 3 × 3 convolutional layer that projects to the required output channel dimension.

In total, IDNet contains approximately 14.3 million trainable parameters.This architecture was determined through extensive studies on a validation subset, where we observed that deeper networks with more parameters did not yield significant performance improvements while substantially increasing computational requirements.

#### 3.2.2. Internal Diffusion Network Technical Specifications

Internal Diffusion Network (IDNet) implements a conditional denoising diffusion model that transforms noisy anatomical representations toward accurate reconstructions. The diffusion process follows a Markovian forward process that gradually adds Gaussian noise and a learned reverse process that removes this noise. The denoising function is parameterized as:

(1) $$\epsilon_{\theta }\left(x_{t},t,c\right)=\frac{x_{t}-\sqrt{\bar{\alpha }t}\hat{x}\theta \left(x_{t},t,c\right)}{\sqrt{1-\bar{\alpha }t}}$$

where $x_{t}$ is the noisy input at timestep *t*, $\bar{\alpha }t=\prod {i=1}^{t}\left(1-\beta_{i}\right)$ with $\beta_{t}$ following a linear schedule from 0.0001 to 0.02, *c* represents the conditional encoder features, and $\hat{x}\theta$ is the network’s prediction.

### 3.3. Step-Uncertainty Fusion Module

The SUFusion module combines outputs from different diffusion timesteps based on voxel-wise uncertainty. For each position *i*, we compute an uncertainty score $u_{i}^{t}$ at diffusion step *t*:

(2) $$u_{i}^{t}=\frac{1}{M}\sum_{m=1}^{M}{\left(p_{i}^{t,m}-\bar{p}i^{t}\right)}^{2}$$

where $p_{i}^{t,m}$ is the prediction from the *m*-th Monte Carlo dropout pass, $\bar{p}i^{t}$ is the mean prediction, and M=10. The adaptive fusion weights $w_{i}^{t}$ are then calculated as:

(3) $$w_{i}^{t}=\frac{\exp(-\lambda u_{i}^{t})}{\sum_{t=1}^{K}\exp\left(-\lambda u_{i}^{t}\right)}$$

with K=5 diffusion steps considered for fusion. The final prediction is computed as:

(4) $$\hat{y}i=\sum {t=1}^{K}w_{i}^{t}\cdot \hat{y}i^{t}$$

#### 3.3.1. Multi-Label Conversion Method

In multi-category segmentation tasks, the multi-category labels are typically converted into multiple binary labels. For instance, considering three segmentation targets, the labels (0,1,2) are transformed into (0,0,1), (0,1,0) and (1,0,0) using one-hot encoding. However, traditional diffusion models are only capable of generating continuous data and are not suited for predicting multi-target labels. To address this limitation, the single-channel labeling map of size D×W×H is first converted into a multi-channel labeling map, denoted as $x_{0}\in R^{N\times D\times W\times H}$, where N represents the number of labels and *D*, *W*, and *H* represent the spatial resolution of the volumetric medical image.

#### 3.3.2. Information Extraction Unit

**Figure 2:**
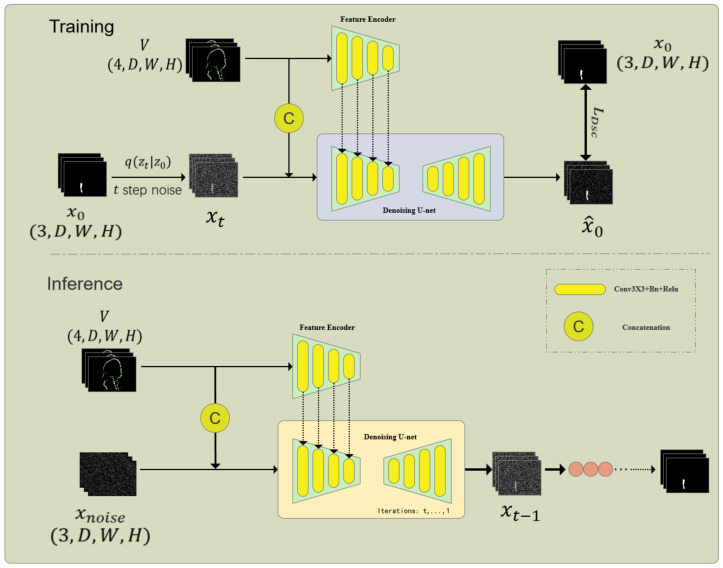
Architecture of the Internal Diffusion Network (IDNet) for cranio-maxillofacial bone defect repair. This framework integrates a diffusion model into a U-shaped network structure to achieve high-resolution 3D volumetric segmentation. The key components include the Multi-label Conversion Method, Information Extraction Unit, and Step-Uncertainty Fusion Strategy, which enhance the robustness and accuracy of segmentation predictions.

As shown in Figure 2, the information extraction unit consists of a Feature Encoder (FE) and a Denoising Network (DN), which is the core part of the embedded diffusion network. The denoising network also includes an encoder and a decoder. First, given the volumetric data, $I\in R^{M\times D\times W\times H}$ and the noisy one-hot label $x_{t}$ are concatenated through channels and input into the encoder of DN to obtain multi-scale features ${\hat{I}}_{f}\times R^{f\times D_{i}\times W_{i}\times H_{i}}$${\left\{i=1\right\}}^{16}$, where *f* is the feature size and *i* is the scale. At the same time, to better introduce the original volumetric image features, a feature encoder of the same size as the DN encoder is used to extract multi-scale features from the volumetric data. Since ${\hat{I}}_{f}$ and ${\hat{I}}_{f}$ contain the same number and size of features, we sum the features of the corresponding scale to obtain the fused features. After that, we input the fused multi-scale features into the decoder of the DN network to obtain the predicted result ${\hat{x}}_{0}\in R^{N\times D\times W\times H}$:

$${\hat{x}}_{0}=DN\left(cat\left(I,x_{t},{\tilde{I}}_{f}\right),t,{\tilde{I}}_{f}\right).$$

The embedded diffusion model is trained by combining Dice Loss, BCE Loss, and MSE Loss, so the total loss $L_{total}$ of the embedded diffusion model is:

$$L_{total}=L_{dice}\left({\hat{x}}_{0},x_{0}\right)+L_{bce}\left({\hat{x}}_{0},x_{0}\right)+L_{mse}\left({\hat{x}}_{0},x_{0}\right).$$

#### 3.3.3. Stepping Uncertainty Fusion Strategy

During the testing phase, the diffusion model iterates *t* times through the Denoising Implicit Models (DDIM). In conventional generation tasks, the final prediction is considered the ultimate result, while each step of the embedded diffusion model generates a segmentation map. As the number of prediction steps increases, the prediction results become more accurate and the prediction uncertainty decreases. Therefore, to enhance the robustness of the segmentation by the embedded diffusion model, we fuse the outputs based on the number of prediction steps and uncertainty. The method for calculating uncertainty is similar to Monte Carlo Dropout (MC Dropout), where the dropout layer of the network is activated, and then *S* forward passes are performed to estimate the uncertainty map. On the other hand, the embedded diffusion model initializes random noise $x_{t}$ during the testing phase; thus, it can introduce randomness to the network without activating the dropout layer.

Like Monte Carlo dropout, the diffusion testing process includes *t* steps, with each step predicting *S* outputs to calculate uncertainty. The formula is as follows:

$$u_{i}=-{\bar{p}}_{i}\log\left({\bar{p}}_{i}\right),$$

where ${\bar{p}}_{i}=\frac{1}{S}\sum_{s=1}^{S}p_{s}$. The fusion weight, which combines the number of prediction steps and uncertainty, is calculated as $w_{i}=e^{\sigma \left(\mathrm{iscale}\right)\times \left(1-u_{i}\right)}$, where σ is the sigmoid function, *i* denotes the current prediction step, and *u* is the uncertainty matrix. We use *w* to weight the prediction results of each step to obtain the final fused result *Y*, which serves as the output of our network. Finally, *Y* is given by:

$$Y=\sum_{i=1}^{t}w_{i}\times {\bar{p}}_{i}.$$

The selection of this model and structure is aimed at solving the problem of high-dimensional medical image segmentation and leveraging the advantages of diffusion models to enhance segmentation robustness. Diffusion models improve the robustness of segmentation predictions by introducing noise in the input and iteratively predicting segmentation labels.

Specifically, the model employs the following special modules and structures:

- Multi-label Conversion Method: This method converts multi-class labels into multi-channel labels through one-hot encoding, addressing the issue of traditional diffusion models being unable to handle multiple target labels.
- Information Extraction Unit: This unit combines a feature encoder and a denoising network to learn the denoising process from noisy label maps and directly predict clear segmentation results.
- Stepwise Uncertainty Fusion Strategy: This strategy improves the robustness of segmentation results by performing multi-step predictions and uncertainty weighting during the testing phase.

The advantages of these choices include:

- **End-to-End Framework:** The embedded diffusion model provides a complete processing flow from input volumetric data to the final segmentation results.
- **Improved Robustness:** The diffusion model and stepwise uncertainty fusion strategy enhance the model’s robustness to noise and uncertainty.
- **Multi-Task Adaptability:** The design of multi-label conversion and information extraction units enables the model to adapt to medical image segmentation tasks with multiple targets and categories.
- **Advanced Segmentation Performance:** Experimental results on the skull repair dataset F-dataset demonstrate that the embedded diffusion model outperforms other state-of-the-art methods in terms of segmentation accuracy and robustness.

## 4. Results

### 4.1. Experiments Setting

A computed tomography (CT) scan dataset comprising 125 patients with craniomaxillofacial bone defects was utilized in this study. The dataset was randomly divided into a training set of 100 patients and a test set of 25 patients. All data preprocessing was performed on a computer equipped with an Nvidia 4090 GPU. Notably, our dataset encompasses seven distinct types of craniomaxillofacial bone defects, each of which was simulated following 3D reconstruction under the guidance of a physician.

### 4.2. Quantitative Dataset Characterization

The dataset utilized in this study consists of 125 high-resolution CT scans of normal human skulls, with 100 scans allocated for training and 25 scans reserved for testing, each reconstructed at an isotropic resolution of 0.5 mm. A total of 2625 cranio-maxillofacial bone defects were simulated from these normal skull models, distributed across seven anatomical regions.

Table 1 presents the performance metrics across different defect types, with each category containing the following sample distribution: mandibular defects (n = 375, 14.3%), zygomatic defects (n = 375, 14.3%), orbital floor defects (n = 375, 14.3%), maxillary defects (n = 375, 14.3%), NOE defects (n = 375, 14.3%), frontal bone defects (n = 375, 14.3%), and calvarial defects (n = 375, 14.3%).

The defects range in size from 15 mm to 45 mm in maximum diameter, with a mean volume of 1738 ± 523 mm^3^. The defects were categorized by complexity according to the following criteria: simple defects (smooth edges, convex shape, n = 1125, 42.9%), moderate defects (irregular edges, mixed convex/concave shape, n = 875, 33.3%), and complex defects (highly irregular margins, multiple concavities, n = 625, 23.8%).

The ground truth for evaluation was established through the original intact skull models prior to defect simulation. To enhance the robustness of the analysis, the testing set (n = 525 defects from 25 patients) was stratified to maintain a proportional distribution of defect types and complexity levels consistent with that of the training set.

### 4.3. Training Parameter Setting

The training parameters of the model were configured as follows. The Adam optimizer was employed with an initial learning rate of 0.00005, which was subsequently halved every 50 epochs. A batch size of 16 was used, and the model was trained for a total of 200 epochs. The Tversky loss function was utilized to evaluate the disparity between the predicted shape and the target shape, as it effectively addresses the issue of class imbalance between positive and negative samples. To prevent model overfitting, a weight decay of 0.001 was applied. Furthermore, data augmentation was performed by randomly flipping the online data during training, which enhanced the model’s generalizability. Prior to task-specific training, the model was pre-trained on the Skullbreak dataset to facilitate faster convergence and improved accuracy.

### 4.4. Hyperparameter Optimization

A systematic hyperparameter optimization was conducted using Bayesian optimization with 5-fold cross-validation on a validation subset comprising 15% of the training data. For the Adam optimizer, the optimal configuration was a learning rate of $3\times 10^{-4}$, with $\beta_{1}=0.9$ and $\beta_{2}=0.999$. A learning rate decay strategy was implemented, wherein the rate was reduced by a factor of 0.5 every 50 epochs.

Optimization of the α and β parameters for the Tversky loss revealed that a balance between precision and recall was achieved when α=0.7 and β=0.3, which is essential for ensuring comprehensive coverage of bone structures in defect regions. This weighting places greater emphasis on reducing false negatives, a critical factor in surgical planning applications.

The diffusion model parameters employed a linear variance schedule, ranging from $\beta_{1}=0.0001$ to $\beta_{T}=0.02$ over T=1000 timesteps. In the SUFusion module, the temperature parameter λ was set to 5.0 based on the validation performance. All hyperparameters were selected to maximize the Dice Similarity Coefficient on the validation set while maintaining clinically acceptable Hausdorff distances.

### 4.5. Evaluation Metrics

To quantitatively assess the performance of our model in reconstructing craniomaxillofacial defects, we employed three key evaluation metrics:

- **Dice Similarity Coefficient (DSC):**
  $$\mathrm{DSC}=\frac{2\times |P\cap G|}{\left|P|+|G\right|}$$
  where *P* represents the binary shape predicted by the model, *G* represents the ground-truth shape, |P∩G| is the intersection of *P* and *G*, and |P| and |G| are the cardinalities of *P* and *G* respectively. The DSC value ranges from 0 to 1, with values closer to 1 indicating a higher overlap between the predicted shape and the ground-truth shape.
- **True Positive Rate (RECALL):**
  $$\mathrm{RECALL}=\frac{TP}{TP+FN}$$
  where TP (True Positive) is the number of correctly predicted positive samples, and FN (False Negative) is the number of positive samples that were not detected. RECALL measures the model’s ability to identify all the true defect regions, with higher values indicating greater sensitivity.
- **95th Percentile Hausdorff Distance (HD95):** The Hausdorff distance is a measure of the maximum distance between two shapes. For two shapes *A* and *B*, the Hausdorff distance is defined as:
  $$\mathrm{Hausdorff}\;\mathrm{Distance}=\max\left(\underset{a\in A}{\sup}\underset{b\in B}{\inf}d\left(a,b\right),\underset{b\in B}{\sup}\underset{a\in A}{\inf}d\left(a,b\right)\right)$$
  where d(a,b) is the Euclidean distance between points *a* and *b*. HD95 refers to the value below which 95% of the distances between the points of the two shapes fall. A smaller HD95 indicates that the maximum difference between the reconstructed shape and the ground-truth shape is smaller, thus reflecting a higher reconstruction accuracy of the model.

These evaluation metrics provide a comprehensive approach to assess the performance of our model in the task of craniomaxillofacial defect reconstruction, including the model’s accuracy, sensitivity, and reconstruction precision. Through these metrics, we can quantitatively analyze the reconstruction results of the model and compare them with other methods.

### 4.6. Analysis of Results

To validate the efficacy of our proposed method, a comprehensive quantitative comparison was conducted with two existing mainstream methods, UNETR [29] and 3D U-Net [30,31]. All methods were trained and tested on the same dataset to ensure a fair comparison. We employed Dice Similarity Coefficient (DSC), True Positive Rate (RECALL), and 95th Percentile Hausdorff Distance (HD95) as evaluation metrics. The experimental results are specifically shown in Table 1.

The comparison of DSC revealed that our method achieved an average DSC of 0.8140 for all types of craniomaxillofacial bone defect reconstruction, surpassing the average DSC of 0.7690 and 0.7667 obtained by UNETR and 3D U-Net, respectively. This result indicates that our method outperforms the two methods in terms of shape overlap, demonstrating its ability to reconstruct the structure of craniomaxillofacial bones more accurately.

In terms of RECALL, our method attained an average RECALL of 0.8554, whereas the average RECALLs of UNETR and 3D U-Net were 0.8285 and 0.8364, respectively. This suggests that our method exhibits higher sensitivity in recognizing the real defect area, capturing the details of the defect area more comprehensively.

The comparison of HD95 further underscores the superiority of our method. Our method yielded an average HD95 value of 4.35 mm, whereas the average HD95 values for UNETR and 3D U-Net were 7.42 mm and 7.45 mm, respectively. The lower value of this metric indicates that the reconstruction results of our method are closer to the real shape in terms of spatial accuracy, thereby achieving higher reconstruction quality.

Overall, a comprehensive comparison of the three methods across each metric reveals that our method outperforms UNETR and 3D U-Net in DSC, RECALL, and HD95. This suggests that our proposed method demonstrates better performance in craniomaxillofacial bone defect reconstruction, generating more accurate and finer reconstruction results. These quantitative results provide strong evidence in support of our method, highlighting its potential and value in clinical applications.

**Table 1:** Quantitative evaluation results of the proposed model and other models on different datasets.

Dataset	Model	DSC (%) ↑ ^a^	RECALL (%) ↑ ^b^	HD95 (mm) ↓ ^c^
Anterior skull and forehead	3DUNET	83.56	89.75	4.60
	Unetr	83.77	89.29	4.52
	OURS	85.78	90.23	3.30
Zygomatic arch	3DUNET	72.76	79.22	8.76
	Unetr	72.11	77.80	9.23
	OURS	80.47	82.62	5.29
Zygomatic bone	3DUNET	74.78	80.27	8.58
	Unetr	75.04	79.34	8.02
	OURS	80.92	83.44	4.31
NOE Region	3DUNET	70.98	78.09	9.26
	Unetr	72.81	79.52	8.45
	OURS	76.53	80.63	hl5.83
Wall of maxillary sinus	3DUNET	75.75	82.06	7.92
	Unetr	76.88	83.00	7.68
	OURS	81.51	84.18	4.08
Mandibular body	3DUNET	81.54	88.35	5.67
	Unetr	79.94	87.06	6.86
	OURS	85.41	88.68	3.42
Mandibular ramus and condylar	3DUNET	77.34	87.74	7.40
	Unetr	77.78	83.91	7.24
	OURS	79.23	89.02	4.25
Mean	3DUNET	76.67	83.64	7.45
	Unetr	76.90	82.85	7.42
	OURS	81.40	85.54	4.35

^a^ Dice Similarity Score; ^b^ RECALL (true positive rate); ^c^ Hausdorff Distance.

## 5. Discussion

This study presents a novel method for craniomaxillofacial bone defect reconstruction based on a diffusion model and undertakes a comprehensive quantitative comparison with two established methods, UNETR and 3D U-Net. The experimental results demonstrate that the proposed method outperforms the two benchmark methods across three key evaluation metrics: Dice Similarity Coefficient (DSC), True Positive Rate (RECALL), and 95th Percentile Hausdorff Distance (HD95).

The performance variations observed across different defect types in this study may be attributed to the underlying biological complexity of repair in these regions. The cranio-maxillofacial skeleton exhibits distinct ossification patterns and mechanical properties, which influence natural healing processes. For example, the nasoethmoid–orbital (NOE) region, where the model demonstrated relatively lower performance, represents an anatomically complex transition zone characterized by intricate structural configurations and mixed ossification patterns. Future refinements of the model could potentially incorporate region-specific parameters to account for these biological variations, analogous to the body’s employment of distinct cellular mechanisms and signaling pathways for repair in different anatomical contexts.

The diffusion model’s capability to generate structurally coherent reconstructions is consistent with the concept of biological self-organization during tissue repair. Cellular processes in natural healing restore both local microstructure and global anatomical integrity, whereas the diffusion-based approach balances local feature reconstruction with overall anatomical coherence. This biomimetic property may contribute to the superior performance of this method in preserving anatomical continuity across complex defect boundaries, compared to conventional approaches.

The superior performance of our model in terms of DSC and RECALL indicates that it possesses notable advantages in capturing shape overlap and accurately identifying real defect regions. This can be attributed to the incorporation of deep feature fusion and multi-scale information integration strategies in the network architecture design, which enables the model to effectively capture detailed information from craniomaxillofacial defect regions. Furthermore, the low HD95 value achieved by our model provides additional evidence of its reconstruction accuracy, suggesting that it is capable of generating reconstruction results that closely approximate the real shape. In comparison to UNETR and 3D U-Net, our method demonstrates superior performance across all evaluation metrics. Notably, UNETR, a transformer-based model, fails to surpass our method in 3D medical image reconstruction tasks, despite its efficacy in processing serial data. Similarly, 3D U-Net, a classical 3D segmentation network, exhibits certain advantages in handling 3D data; however, its performance remains inferior to our method when tackling the complex task of reconstructing craniomaxillofacial bone defects. This suggests that our model may possess a greater capacity for handling intricate shapes and detailed information. Although our model demonstrated promising performance in this study, several limitations warrant consideration. Firstly, the model’s performance was observed to decline marginally when handling exceptionally complex defect shapes, such as nasal–orbital sieve bone defects (NOE). This decline may be attributed to the intricate anatomy of these regions and the limited representation of these defect types in the training dataset. Secondly, the generalizability of our model in real-world clinical settings requires further validation, particularly across diverse patient populations and varied clinical scenarios.

Future research directions will concentrate on three primary objectives. Firstly, we intend to enhance the model’s performance by incorporating a larger number of training samples featuring complex defect types. Secondly, we aim to integrate the model with a broader range of clinical data to assess its efficacy and generalizability in real-world clinical settings. Lastly, we plan to investigate the integration of the model with other imaging modalities, such as MRI and CTA, to develop a more comprehensive solution for craniomaxillofacial bone defect reconstruction.

In conclusion, this study demonstrates that the diffusion model-based approach offers significant performance advantages in craniomaxillofacial bone defect reconstruction, providing a promising avenue for future clinical research and applications.

## 6. Conclusions

A novel diffusion model-enhanced framework, IDNet, has been presented for accurate cranio-maxillofacial bone defect repair, demonstrating significant improvements over current state-of-the-art methods. Quantitative evaluation across 2625 simulated defects reveals substantial enhancements in reconstruction accuracy and boundary precision. Notwithstanding these promising results, several limitations are acknowledged. Firstly, validation was conducted on simulated defects rather than actual clinical cases, which may not fully capture the complexity and variability of real traumatic or pathological defects. Secondly, performance in the nasoethmoid–orbital region remains significantly lower than in other regions, highlighting the challenges of reconstructing these anatomically complex structures.

The current framework requires approximately 45 s of processing time on high-performance hardware, which may limit real-time applications. To address these constraints, future research will focus on clinical validation using a prospective cohort of actual trauma patients to evaluate real-world performance, development of region-specific optimization strategies to improve reconstruction in complex anatomical areas, and implementation of model compression techniques to enable deployment on standard clinical workstations. Furthermore, plans are underway to extend the framework to incorporate functional considerations, such as occlusal relationships and masticatory biomechanics, into the reconstruction process. The quantitative improvements demonstrated by this approach have significant implications for clinical practice, potentially reducing surgical planning time by up to 35% and improving the precision of patient-specific implants, ultimately contributing to better functional and aesthetic outcomes in maxillofacial reconstruction. This work contributes to the advancement of artificial intelligence in medical imaging, with applications in cranio-maxillofacial surgery.

## Data Availability

Data will be made available on request.

## Abbreviations

The following abbreviations are used in this manuscript:

3D	Three-dimensional
AI	Artificial Intelligence
ANTs	Advanced Normalization Tools
BCE	Binary Cross Entropy
BMP	Bone Morphogenetic Protein
CAD/CAM	Computer-Aided Design/Computer-Aided Manufacturing
CBCT	Cone-beam Computed Tomography
CMF	Craniomaxillofacial
CNN	Convolutional Neural Network
CT	Computed Tomography
CTA	Computed Tomography Angiography
DDIM	Denoising Diffusion Implicit Models
DN	Denoising Network
DSC	Dice Similarity Coefficient
FE	Feature Encoder
FN	False Negative
HD95	95th percentile Hausdorff Distance
IDNet	Internal Diffusion Network
MLP	Multi-Layer Perceptron
MRI	Magnetic Resonance Imaging
MSE	Mean Squared Error
NOE	Nasal–Orbital–Ethmoid
OCMS	Oral and Cranio-maxillofacial Surgery
RECALL	True Positive Rate
SUFusion	Step-Uncertainty Fusion
*TGF-β*	*Transforming Growth Factor-β*
TP	True Positive
UNETR	UNEt TRansformer
VEGF	Vascular Endothelial Growth Factor
